# Analysis of the *trap* gene provides evidence for the role of elevation and vector abundance in the genetic diversity of *Plasmodium relictum* in Hawaii

**DOI:** 10.1186/1475-2875-11-305

**Published:** 2012-09-03

**Authors:** Margaret E M Farias, Carter T Atkinson, Dennis A LaPointe, Susan I Jarvi

**Affiliations:** 1 Department of Pharmaceutical Sciences, College of Pharmacy, University of Hawaii at Hilo, Hilo, HI, 96720, USA; 2 US Geological Survey, Pacific Island Ecosystems Research Center, Hawaii National Park, HI, 96718, USA

**Keywords:** *Plasmodium relictum*, *trap*, SNP, Amakihi, Diversity, Hawaiian honeycreepers, Mosquitoes

## Abstract

**Background:**

The avian disease system in Hawaii offers an ideal opportunity to investigate host-pathogen interactions in a natural setting. Previous studies have recognized only a single mitochondrial lineage of avian malaria (*Plasmodium relictum*) in the Hawaiian Islands, but cloning and sequencing of nuclear genes suggest a higher degree of genetic diversity.

**Methods:**

In order to evaluate genetic diversity of *P. relictum* at the population level and further understand host-parasite interactions, a modified single-base extension (SBE) method was used to explore spatial and temporal distribution patterns of single nucleotide polymorphisms (SNPs) in the thrombospondin-related anonymous protein (*trap*) gene of *P. relictum* infections from 121 hatch-year amakihi (*Hemignathus virens*) on the east side of Hawaii Island.

**Results:**

Rare alleles and mixed infections were documented at three of eight SNP loci; this is the first documentation of genetically diverse infections of *P. relictum* at the population level in Hawaii. Logistic regression revealed that the likelihood of infection with a rare allele increased at low-elevation, but decreased as mosquito capture rates increased. The inverse relationship between vector capture rates and probability of infection with a rare allele is unexpected given current theories of epidemiology developed in human malarias.

**Conclusions:**

The results of this study suggest that pathogen diversity in Hawaii may be driven by a complex interaction of factors including transmission rates, host immune pressures, and parasite-parasite competition.

## Background

The avian disease system in Hawaii is complex and involves interactions between susceptible native birds (e.g. the Hawaiian honeycreepers), resistant introduced birds, mosquitoes and pathogens, including the potential relationship between avian malaria (*Plasmodium relictum*) and avian pox (*Avipoxvirus*) [1-4]. Dynamics of the disease system vary across the Hawaiian landscape, where malaria and pox transmission is year-round and endemic at low-elevations (<300 m above sea level), but follows a highly seasonal pattern at mid-elevation (1,000 to 1,300 m) [5]. With a range of susceptible and resistant hosts, varying levels of transmission, and a limited number of pathogens, the avian disease system in Hawaii offers an ideal opportunity to investigate many aspects of host-pathogen and pathogen-pathogen interactions in a natural setting.

Much of the current knowledge of avian malaria in Hawaii is restricted to results of experimental infections under controlled conditions, epidemiological studies of prevalence and transmission in wild populations, and recovery and necropsy of moribund and dead forest birds [6]. Although Beadell and colleagues [7] have identified *P. relictum* in Hawaii as a single mitochondrial lineage (GRW4 [8]), cloning and sequencing studies have found diversity in nuclear genes, including *trap*[9].

The *trap* gene of *Plasmodium* encodes the thrombospondin-related anonymous protein (TRAP), a surface protein that has been implicated in host immune system evasion as well as movement through the vector and invasion of host cells [10-12]. The *trap* gene has been shown to be under diversifying selection in the human malaria parasites *Plasmodium falciparum*[13] and *Plasmodium vivax*[14] and appears to be highly variable in *P. relictum* in Hawaii [9]. Understanding how *trap* alleles are distributed throughout the host population may help us to understand how factors, such as transmission rates, selective pressure from the host immune system, and within-host competition, drive genetic diversity of malaria parasites. For instance, if surface protein gene diversity is driven by selective pressure from the host immune system or by varying transmission rates [15], prevalences of *trap* polymorphisms might be expected to differ between locations with genetically distinct host populations or differing transmission rates. Because higher transmission rates may allow greater mutation and recombination between parasites, current theories of epidemiology in malaria predict higher parasite diversity in areas of higher transmission, especially in areas where transmission is endemic [15]. However, such theories have never been applied to the disease system in Hawaii.

Until now, all genetic studies of *trap* and other nuclear genes of *P. relictum* in Hawaii have focused on a small number of individual hosts. To further explore population-level genetic variation in *P. relictum* in Hawaii, an SBE approach was employed to determine the spatial distribution of alleles at eight SNP loci in the *trap* gene of *P. relictum* infecting Hawaii amakihi (*Hemignathus virens*, hereafter amakihi). Genotype information was collected from a large number of individual blood samples collected from various sites and elevations on the east side of Hawaii Island and then analysed using logistic regression to investigate the factors driving parasite diversity in this system.

## Methods

**Figure 1:**
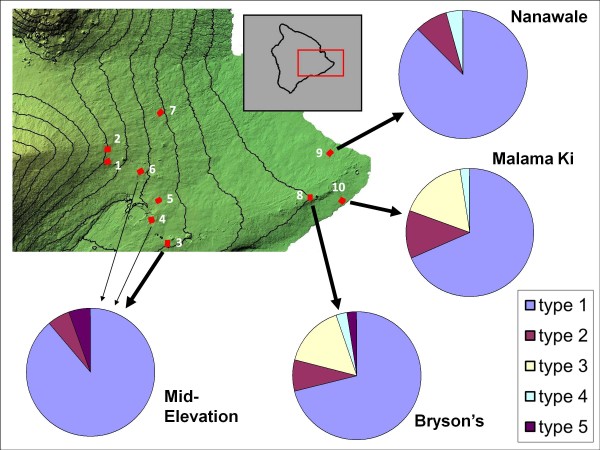
**Distribution of composite trap genotypes by study site map of eastern Hawaii Island showing locations of 1 sq km study sites (red squares).** Solid lines indicate 250 m elevational contours. High-elevation sites are (1) CJ Ralph and (2) Solomon’s, mid-elevation sites are (3) Ainahou Ranch, (4) Crater Rim, (5) Cooper’s, (6) Puu Unit and (7) Waiakea, and low-elevation sites are (8) Bryson’s, (9) Nanawale, and (10) Malama Ki.

Sample collection for this study was conducted as part of a larger investigation of the ecology of avian malaria on the windward slopes of Mauna Loa and Kilauea Volcanoes (NSF DEB 0083944). Collection of field samples was approved by the University of Hawaii Institutional Animal Care and Use Committee, protocol 00-035. Native and non-native forest birds were captured with mist nets on a monthly sampling schedule (minimum two to four days/month/site) at nine 1 sq km study sites in 2002, 2003 and 2004 [16]. Two of these sites (Figure 1, CJ Ralph and Solomon’s) were located at high-elevation (>1,500 m above sea level), four (Crater Rim, Cooper’s, Puu Unit and Waiakea) at mid-elevation (1,000 to 1,300 m), and three (Bryson’s, Nanawale and Malama Ki) at low-elevation (<300 m). Birds were measured, sexed, and aged, and a blood sample was obtained by jugular venipuncture. To increase sample size, this study also included blood samples concurrently collected from a fifth, mid-elevation site, Ainahou Ranch.

The amakihi was chosen as the focus of this study due to its significant numbers at low-elevation in comparison to other native species [16]. Furthermore, the amakihi is thought to be a more sedentary species [17], reducing the possibility of a bird becoming infected away from the study site. To eliminate the possibility of sampling a bird that was infected in a prior year, only hatch-year amakihi were considered. All amakihi captured were tested for infection with *P. relictum* using a combination of serological methods and microscopy [16], which has been shown to be the best approach for maximizing detection of infection [18], and only those shown to be infected were included in this study.

Procedures used to sample and dissect adult mosquitoes from these sites have been previously described [16]. Briefly, modified Center for Disease Control (CDC) miniature light traps baited with 300 g of dry ice (CO_2_) (hereafter referred to as CO_2_ traps) were operated from December 2001 to December 2004 to sample adult mosquito populations at the ten sites described above. CO_2_ traps were operated without lights and hung in the forest canopy at a height of 4–12 m. CO_2_ traps were placed at 25 stratified, random locations within each 1 sq km field site. Traps were operated at each site monthly, from mid-afternoon to mid-morning for four consecutive days. At the Ainahou Ranch site, CO_2_ traps were operated at 21 fixed locations for four consecutive days every other week. Trap captures were collected every day and mosquitoes were counted, identified, and brought to the laboratory for dissection. Malarial infection was determined by phase-contrast microscopy (400X) of midguts and salivary glands for oocysts and sporozoites, respectively; mosquitoes were considered infected if either oocysts and/or sporozoites were detected. Only results from *Culex quinquefasciatus*, the recognized vector of avian malaria in Hawaii [19], were included in the analysis.

Genetic diversity of *P. relictum* in each host was measured by determining allele prevalence at each of eight *P. relictum trap* SNP loci using the nucleotide-constrained SBE method described by Farias and Jarvi [20]. The SBE method of SNP genotyping is similar in concept to a Sanger sequencing reaction, except that only dideoxy-nucleotides (ddNTPs) are used. Interrogation primers are designed to anneal to the target sequence one nucleotide upstream of each SNP site. The complementary labelled ddNTP is then incorporated into the primer, terminating further extension and allowing discrimination of the nucleotide(s) present at that locus via capillary electrophoresis and laser detection. As in direct sequencing, multiple alleles at a given locus appear as mixed peaks in the electropherogram. Multiplexing is achieved by adjusting primer length using poly-A or poly-T tails at the 5’ end of each primer [21,22]. A standard SBE reaction usually contains a premix of all four ddNTPs, reaction buffer, enzyme, template and interrogation primers. In the modified nucleotide-constrained approach, removal of the ddNTP corresponding to the common allele from the reaction eliminates amplification of the common allele, allowing improved detection of rare alleles at each locus [20].

Genomic DNA (gDNA) was extracted from infected amakihi blood samples using the DNeasy Tissue Extraction Kit (Qiagen, Valencia, CA, USA) following the manufacturer’s protocol for animal tissues. The *trap* gene of *P. relictum* was then amplified using a nested polymerase chain-reaction (PCR) technique modified from Jarvi *et al.*[9]. In the first reaction, 3–5 μl gDNA were added to a mixture containing 1X reaction buffer, 1.6 mM MgCl_2_, 900 μM total dNTPs, 15 pmol each primers P1 and P2 [18] and 1.25 units FastStart High Fidelity Enzyme Blend (Roche Applied Science, Indianapolis, IN, USA) and subjected to heating to 94°C for 4 min, followed by 25 cycles of 94°C for 30 sec, 50°C for 1 min and 70°C for 2 min, with a final extension step of 70°C for 7 min. Three μl PCR product were then used as template in a nested reaction containing 1X reaction buffer, 1 mM MgCl_2_, 600 μM total dNTPs, 20 pmol each primers P5 and P6 [18], 4% DMSO and 1.25 units of Fast–Start High Fidelity Enzyme Blend. Cycling was as described for the P1/P2 reaction except that the annealing temperature was 60°C and the number of cycles was increased to 35. Purified PCR (QIAquick Gel Extraction Kit, Qiagen) product from each blood sample was used as template in one standard and two modified SBE reactions, with sample analysis and peak detection on the CEQ 8000 capillary electrophoresis system (Beckman-Coulter, Fullerton, CO, USA) as previously described [20]. Following preliminary scoring, an individual bird was labelled as harbouring a potential mixed infection if multiple peaks were observed at any single locus in the standard reaction, or if any peaks were visible in either of the modified reactions. Rare alleles and mixed infections were confirmed by repeated testing from the gDNA level. Mixed or rare peaks not confirmed by a second reaction were subjected to a third round of amplification, purification and genotyping, and a mixed or rare peak was considered valid if it appeared in two of three repeat reactions for a single individual. Ambiguous peaks (heights less than 500 rfu) were considered valid if they appeared in all three repeat reactions.

Data for prevalence of alleles were analysed using a presence/absence approach. Each bird was scored as “nucleotide 1”, “nucleotide 2” or “mixed” for each dimorphic SNP loci. Locus 687 is trimorphic and was scored as “nucleotide 1”, “nucleotide 2”, “nucleotide 3” or “mixed”. Scores from the standard and modified SBE reactions were pooled such that a bird was considered “mixed” at a given locus if the rare peak for that locus appeared in the modified reaction, even if it was absent in the standard reaction. For locus 1293, individuals were scored as “G-only” if a G peak was present in the standard reaction and no C peak was detected in the modified reaction, and “C-only” if these conditions were reversed. An individual was scored as “mixed” at locus 1293 if both G and C peaks were present in the standard reaction or if the second peak was detected in the modified reaction.

Scores from loci in which rare alleles and/or mixed infections were detected were pooled to create a composite infection genotype for each individual (Table 1). In the case of birds captured multiple times, only the earliest blood sample from which parasite DNA could be amplified was used in the statistical analysis. To determine the relationship between composite genotype and a number of predictor variables, composite genotypes were condensed into binary response scores. The presence or absence of the C-allele at locus 1293 was investigated by assigning a score of “1” to individuals infected with the C-allele (genotypes 2 and 3) and a score of “0” to individuals with only the G-allele at locus 1293 (genotypes 1, 4 and 5). Binary responses were then fit via logistic regression to models incorporating the predictor variables of Days (number of days of potential host exposure to infection assuming a hatch date of January 1 of the year of capture), Mosquito Capture (mean number of *Cx. quinquefasciatus* captured in a trap-night averaged across all trap-nights at a site during the estimated lifetime of the bird), Mosquito Infection (mean prevalence of *P. relictum* in captured mosquitoes averaged across all trap-nights at a site during the estimated lifetime of the bird), Elevation (mid or low, treated as a categorical variable), and Year (of host sampling). To improve normality of predictor variables, data were fitted using the log of both Mosquito Capture and Infection. The regression was also repeated using only individuals captured at low-elevation, with Site as an additional categorical predictor variable. The best-fit model for each regression was determined by forward and reverse regression using the Akaike Information Criterion (AIC) to guide model selection. Selected models were further evaluated using goodness of fit tests. Significance of each term in the best model according to AIC was evaluated using Wald’s statistics (data not reported) and analysis of deviance in deletion tests.

**Table 1:** **Composite *trap *genotypes**

**Genotype**	**Nucleotide(s) at 539**	**Nucleotides(s) at 1178**	**Nucleotide(s) at 1293**	***N***
1	T	A	G	92
2	T	A	C	11
3	T	A	G/C	13
4	T/C	A	G	3
5	T	A/G	G	2

Composite *trap* genotypes based on standard and modified single base extension reactions for loci 539, 1178 and 1293. The remaining five loci were monomorphic in this study. *N*, overall number of hatch-year amakihi infected with each composite *trap* genotype.

## Results

Amplification of the focus fragment of the *trap* gene was attempted from 169 hatch-year amakihi samples identified as infected with *P. relictum* based on serology and/or microscopy, and 130 successful PCR products were obtained. Five of these samples represented recapture events, and four other birds were subsequently eliminated from analysis due to problems with re-amplification for repeat reactions. The final number of individuals included in this study was 121 hatch-year amakihi.

Locus 1293 was the only locus found to be polymorphic in the standard SBE reaction; all other loci were monomorphic. Of the 121 amakihi tested, 13 (10.7%) were scored as C-only, 10 (8.3%) were scored as mixed, and the remaining 98 (81.0%) were scored as G-only. Application of the more sensitive modified SBE reactions for detection of rare alleles revealed mixed infections at locus 1293 in three additional individuals, at locus 539 in three individuals and at locus 1178 in two individuals. SNP analysis using the modified SBE approach detected a high number of potential mixed infections at several loci (data not shown), but many of these polymorphisms could not be confirmed through repeat reactions. All unconfirmed mixed infections were scored as single infections in this analysis and all loci other than 539, 1178 and 1293 were considered monomorphic. Because the extent of linkage between these loci is unknown, all loci were treated as linked and scores for loci 539, 1178 and 1293 were used to generate composite infection genotypes for each hatch-year amakihi (Table 1).

The number of individuals analysed and distribution of composite genotypes at each study site is shown in Figure 1. Because data were collected from only a single bird each at the Puu Unit and Crater Rim sites, these sites were merged with Ainahou Ranch under the category of Mid-Elevation. Due to low amakihi capture rates and relatively low infection rates, data were not collected from individuals from Cooper’s, Waiakea, CJ Ralph, or Solomon’s; these sites were therefore not included in further analysis. Single infections with a G at locus 1293 (genotype 1) made up 80.2% of all infections. Distribution of the less common genotypes was not even, with low-elevation sites appearing to be more diverse than sites at mid-elevation. Additionally, all five genotypes were observed at low-elevation, while genotypes 3 and 4 appear absent from mid-elevation. The proportion of birds infected with the C allele at locus 1293 (genotypes 2 and 3) was 22.3% at low-elevation and 5.6% at mid-elevation.

Logistic regression was employed to determine whether the presence or absence of certain alleles in an individual was related to elevation, site, mosquito capture rate, prevalence of infection in mosquitoes, potential days of exposure, or year of capture. Genotypes 4 and 5 were not considered as response variables in separate regressions due to the low numbers of individuals infected with these genotypes. Regressions were therefore performed for only the presence (genotypes 2 and 3) or absence (genotypes 1, 4 and 5) of the C allele at locus 1293.

**Figure 2:**
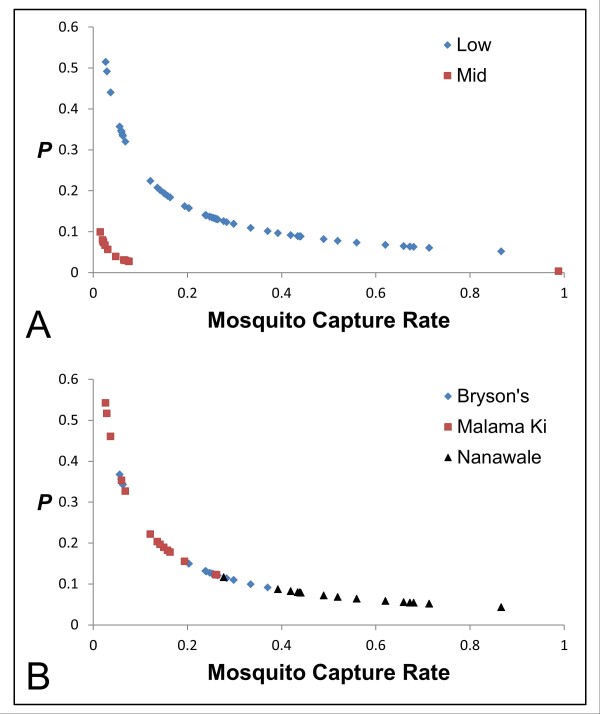
**Predicted probability of infection with genotype 2 or 3.** Predicted probability of infection (*P*) with genotype 2 or 3 based on the best linear regression model plotted as a function of Mosquito Capture Rate (**A**) over all sites and (**B**) over low-elevation sites only.

The predicted likelihood of an individual carrying an infection of genotype 2 or 3 is shown in Figure 2A. The data for this regression were best fitted by a model including log (Capture) (*p* = 0.0007) and Elevation (*p* = 0.0019) (goodness of fit test, *p* = 0.478). The probability of infection with genotype 2 or 3 was higher at low-elevation. Within each elevation, the probability of infection with these genotypes decreased as mosquito capture rate increased. A regression for the likelihood of infection with genotype 2 or 3 at only low-elevation revealed a highly significant effect of log (Capture) (*p* = 0.0003) (goodness of fit test, *p* = 0.97), with the probability of infection with these genotypes again decreasing as mosquito capture rate increased (Figure 2B). Mosquito Infection and Year were included as predictor variables in the full models, but did not appear in either of the final models.

To determine the impact of time on the genotypes of infections, SNP analysis was applied to blood collected at recapture for five of the individuals included here. Time between sampling ranged from one to four months. Four individuals were shown to have the same *trap* genotype at both sampling times, with three individuals infected with genotype 1 and one individual infected with genotype 3 at both captures. In the fifth individual, *trap* genotype 2 was detected in the first sample and *trap* genotype 1 was detected in a sample collected four months later.

## Discussion

Investigating the genetic diversity and distribution of *P. relictum* is a critical component in gaining a better understanding of the avian disease system in Hawaii and may provide insight into how natural processes drive genetic diversity of human malaria as well. According to current theories of malaria epidemiology, pathogen diversity is expected to be highest in areas of high and endemic transmission, and may also be driven by selective pressure from the host immune system and competition between parasite genotypes infecting the same host [15]. Prevalence of malaria infection in both amakihi and mosquitoes is highest at lower-elevation sites [16,23], and low-elevation amakihi have been shown to be more tolerant of *P. relictum* (Atkinson, unpubl data) and genetically distinct from mid- and high-elevation amakihi [24,25] (Jarvi, unpubl data). This study provides the first documentation of diversity in a nuclear gene of *P. relictum* at the population level in Hawaii and for the first time allows the comparison of genetic diversity of *P. relictum* in Hawaii to patterns predicted by epidemiologic theory.

The modified SBE approach to SNP analysis allowed for more sensitive detection of rare alleles than possible with standard SBE or direct sequencing. Mixed infections were detected in 29 of 121 (24.0%) birds. This was a lower prevalence of mixed infections than expected given previous results [9], but is likely a reflection of differences in technique (SNP detection *vs* cloning and sequencing) as well as host age ranges (juveniles in this study *vs* adults in the previous study). Similarly, five of eight expected polymorphic loci were found to be monomorphic in the current sample set. It is possible that the rare alleles at these loci persist in the population at levels too low to be detected by the current method, or are more commonly found in adult birds. Given these factors and the high number of unconfirmed mixed infections observed, the diversity reported here is likely an underestimate of the levels of diversity in the *trap* gene of *P. relictum* in Hawaii.

Of the 121 birds in this sample set, 80.2% were scored as G-only at locus 1293 (genotypes 1, 4 and 5). This is consistent with the results of previous work in individuals with infections originating from Hawaii Island [9]. The current study also documents the first evidence of mixed infections at locus 1293 (genotype 3), with 10.7% of the birds scored as “mixed” for this site. The prevalence of 1293 C-only infections (genotype 2, 9.1%) was close to that of mixed infections and consistent with low detection of 1293 C-only infections on Hawaii Island in the previous study [9].

Logistic regression analysis of possible epidemiological factors revealed elevation to be highly significant in modelling the probability of infection with genotype 2 or 3. While the accuracy of the model may be questioned in light of the disparate sample sizes between mid- (*n* = 18) and low-elevations (*n* = 103), logistic regression is based on proportions rather than raw numbers [26]. The lower sample size at mid-elevation may lead to a less precise estimation of proportions at this elevation, but the inclusion of every available sample in this study means that the data presented here is the best possible estimation of genotype prevalences at both mid- and low-elevation, and the low *p*-value for elevation (*p* = 0.0019) in the regression supports this argument. The results of the regression are not surprising given the large difference in the proportion of birds infected with genotype 2 or 3 between elevations; 22.3% of infections at low-elevation were genotype 2 or 3, compared to only 5.6% of infections at mid-elevation. The role of elevation in the model shows that a bird is more likely to be infected with these genotypes at low-elevation sites and is highly consistent with expectations of greater parasite diversity in areas of higher transmission [15]. While not surprising, this is the first time that a genetic difference between parasite populations has been demonstrated between low-elevation areas of endemic transmission and mid-elevation areas of seasonal transmission in Hawaii.

Perhaps the most interesting and unexpected result in this study is the relationship between probability of infection with genotype 2 or 3 and mosquito capture rate. In theory, a higher mosquito capture rate should reflect higher abundance of mosquitoes and higher transmission of malaria [4]. Thus, higher mosquito capture rates would be predicted to be related to higher likelihood of infection with rare alleles. In the current study, however, logistic regression showed that the likelihood of infection with a rare *trap* allele in Hawaii decreased as the mosquito capture rate increased. This counterintuitive relationship had the greatest statistical significance in the model predicting the likelihood of infection with genotype 2 or 3 in low-elevation birds only (Figure 2B). It is interesting to note that while mosquito capture rate was an important component of all regressions in this study, prevalence of *P. relictum* in captured mosquitoes did not appear in any of the final models and thus does not appear to influence the likelihood of infection with genotype 2 or 3.

It is possible that the inverse relationship between mosquito capture rates and probability of infection with rare genotypes reflects the correlation of mosquito capture rates with another variable not tested here. For instance, higher capture rates in attractive mosquito traps could be linked to lower host density, since traps will be less attractive to mosquitoes when live hosts are present in higher densities. Since lower host densities may lead to reduced transmission, in this scenario, lower parasite diversity might be expected even with increased mosquito capture rates. This may be especially true at Nanawale, the site with the highest mosquito capture rates but lowest mean amakihi capture rates during the course of sample collection for this study [16].

Another intriguing possibility is that the decrease in likelihood of infection with a rare allele observed here is evidence for within-host competition between genetic variants of *P. relictum* in Hawaii. Based on the role of elevation in the best models, it can be assumed that a certain baseline level of endemic transmission is required for increased proliferation of rare alleles. As transmission increases, individual birds are exposed to a greater number of infective bites, and have greater opportunity to acquire additional genetic variants. If all variants are equally competitive, higher numbers of mixed infections would be expected as transmission increases. If, however, genotype 1 is able to out-compete genotypes 2 and 3, the ratio between genotype 1 and the rare genotypes within an individual could become highly skewed, preventing the detection of the rare genotypes and making it appear that infection with genotype 2 or 3 is less likely as transmission increases. Support for this possibility can be found in the re-captured individual that was initially infected with genotype 2, but found to be infected with genotype 1 four months later. Experimental infections and long-term studies have shown that once infected, amakihi remain chronically infected for life [18] (Atkinson, unpubl data), thus this bird would not be expected to have completely cleared parasites with genotype 2 from its system prior to re-infection with genotype 1. It is therefore suspected that this individual actually harbours a mixed infection at *trap* locus 1293 (genotype 3), with the 1293-C parasites present at levels below the detection limit of this study at the time of the second capture. Whether the apparent competitive advantage and increased proliferation of genotype 1 over genotype 2 in this individual is the result of order of infection, increased fitness of genotype 1, or differing interactions of the genotypes with the host immune system is unknown, but one of these mechanisms may be the driving force in the increased likelihood of infection with genotype 1 as mosquito capture rates increase.

## Conclusions

This study represents the first documentation of diversity in a nuclear gene of *P. relictum* at the host population level in Hawaii. The majority of individuals (76%) were infected with a single *trap* genotype with no rare alleles (genotype 1). Likelihood of infection with genotype 2 or 3 was higher at low-elevation, and decreased as mosquito capture rates increased. Demonstrating the links between parasite diversity, elevation, and vector capture rates in this system is an important step in understanding the potential impacts of transmission rates, host immune pressures, and within-host competition on pathogen diversity. Further studies using 454 amplicon sequencing of multiple parasite genes from a larger host sample set are in progress and are expected to yield additional insights.

## Abbreviations

AIC: Akaike information criterion; gDNA: Genomic DNA; SBE: Single base extension; SNP: Single nucleotide polymorphism; TRAP: Thrombospondin-related anonymous protein.

## Competing interests

The authors declare that they have no competing interests.

## Authors’ contributions

MEMF participated in the design of the study, carried out the genotyping studies, performed the statistical analyses and drafted the manuscript. CTA carried out diagnostics on all avian blood samples. DAL carried out all of the mosquito studies. SIJ conceived of the study, participated in its design and coordination and oversaw the drafting of the manuscript. All authors assisted in editing early drafts and read and approved the final manuscript.
